# The Specificity and Reliability of Conflict Adaptation: A Mouse-Tracking Study

**DOI:** 10.3389/fpsyg.2021.770509

**Published:** 2022-01-11

**Authors:** John G. Grundy

**Affiliations:** Department of Psychology, Iowa State University, Ames, IA, United States

**Keywords:** conflict adaptation, conflict monitoring, sequential congruency effect, Stroop, flanker

## Abstract

Researchers have recently begun to question the specificity and reliability of conflict adaptation effects, also known as sequential congruency effects (SCEs), a highly cited effect in cognitive psychology. Some have even used the lack of reliability across tasks (e.g., Flanker, and Stroop) to argue against models of cognitive control that have dominated the field for decades. The present study tested the possibility that domain-general processes across tasks might appear on more sensitive mouse-tracking metrics rather than overall reaction times. The relationship between SCE effects on the Stroop and Flanker tasks were examined for the first time using a mouse-tracking paradigm. Three main findings emerged: (1) Robust SCEs were observed for both the Stroop and Flanker tasks at the group level, (2) Within-task split-half reliabilities for the SCE across dependent variables were weak at best and non-existent in many cases, and (3) SCEs for the Flanker and Stroop tasks did not correlate with each other for overall reaction times, but did show significant correlations between tasks on more dynamic measures that captured processes before response execution. These findings contribute to the literature by highlighting how mouse-tracking may be a fruitful avenue by which future studies can examine the specificity and reliability of conflict adaptation and tease apart different theoretical models producing the effects.

## Introduction

**FIGURE 1:**
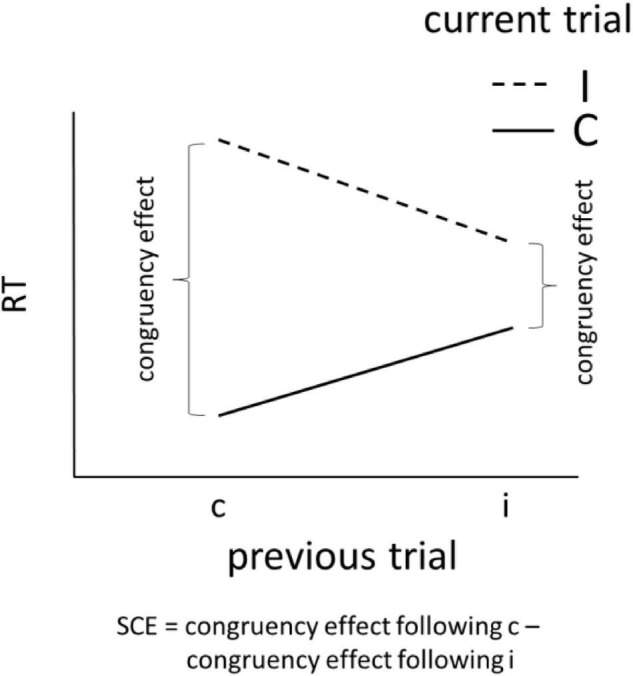
The sequential congruency effect (SCE), a.k.a. conflict adaptation.

The way that we adjust behaviors to events in our day-to-day lives depends largely on a history with recent events. We would not, for instance, be surprised by a firework going off on New Year’s Day after recently hearing several other fireworks exploding. We would, however, likely be shocked by a firework going off on a random Tuesday evening. Human beings adjust responses to these events accordingly—we might jump in response to the second scenario, but not the first. In the laboratory, we observe history-based adjustments in the form of smaller congruency effects following recently encountered incongruent than congruent trials (Gratton et al., 1992) on conflict tasks such as Stroop (1935) and Flanker tasks (Eriksen and Eriksen, 1974; see Figure 1).

This phenomenon, known as “conflict adaptation,” the “Gratton effect,” “congruence sequence effect,” or “sequential congruency effect” (SCE) is a highly cited phenomena in cognitive psychology and continues to spark intense debates regarding its appearance. Most studies have been concerned with the presence or absence of the effect under various conditions as it can inform whether the effect is driven by top-down (Botvinick et al., 2001; Clayson and Larson, 2011; Feldman and Freitas, 2016), bottom-up (Hommel et al., 2004; Schmidt and De Houwer, 2011), or a combination of both top-down and bottom-up processes (review in Egner, 2014).

Several recent studies have begun to test the specificity and reliability of SCEs, given that there are important theoretical implications for debates regarding the underlying mechanisms producing the effects. For example, the conflict monitoring theory (Botvinick et al., 2001) is by far the most influential theory explaining sequential congruency effects (SCEs) and continues to influence new research (e.g., Borsa et al., 2018; Shapira-Lichter et al., 2018; Ho et al., 2019; Mengke et al., 2021). According to the theory, the anterior cingulate cortex (ACC) detects conflict on incongruent trials and recruits the dorsolateral prefrontal cortex (dlPFC) to enhance goal-directed performance. Responding to another incongruent (conflict) trial immediately afterward is easier and more rapid because of this enhancement, explaining the smaller congruency effect following incongruent than congruent trials. Central to the theory, however, is the idea that a common control mechanism drives performance across tasks like the Stroop, Simon, and Flanker, and research continues to accept this premise (Nigbur et al., 2011; Cavanagh and Frank, 2014; Feldman and Freitas, 2016). Accordingly, SCEs should correlate across tasks. Whitehead et al. (2019) recently showed that SCEs were uncorrelated across the Stroop, Simon, and Flanker tasks in three experiments, a finding that challenges the conflict monitoring theory and a domain-general mechanism contributing to SCEs. They also observed that within-task reliability for SCEs was virtually non-existent. However, for both observations, it is possible that the measures they used (button presses) were not sensitive enough to detect shared processes within and between the tasks.

Mouse-tracking might be particularly sensitive to determining the specificity and/or generality of SCEs across tasks because it allows one to examine several cognitive processes that occur between the time that movement is initiated and response completion. Typical button presses only allow a single index of performance, whereas mouse-tracking reveals initiation times, movement times, and dynamic deviations that unfold over the course of a single trial (Freeman and Ambady, 2010; Stillman et al., 2018). Overall *Reaction Time* is the total time from click of a start button to click of the response box. *Initiation Time* is the time from click of the start button to initiation of mouse movement. *Movement Time* is the time after Initiation to completion (click) of response. *Maximum Deviation* is the distance of maximum deviation from a straight line connecting the start point to the correct response box. Large maximum deviations are observed more often on incongruent trials than congruent trials because participants often start moving toward the incorrect response and overcorrect. *Time to Maximum Deviation* is the time from the beginning of the trial that it takes to reach the maximum deviation point. *Area Under the Curve* (AUC) is the area under the curve of the mouse movement trajectory relative to a straight line connecting the start point to the correct response. It is possible that SCEs are domain-general for some of these processes but not others.

**FIGURE 2:**
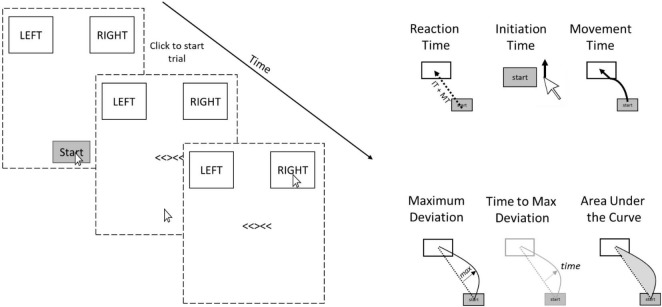
Example trial and dependent variables recorded (to the right) for the Flanker task in the present experiment. The dotted lines represent the contour of the computer screen. The Stroop task was similar but participants responded to the ink of colored words rather than arrows and the response options were RED/GREEN and ORANGE/BLUE rather than LEFT and RIGHT.

Scherbaum and Dshemuchadse (2020) used mouse-tracking versions of the flanker and Simon tasks and demonstrated that the flanker task showed larger interference effects than the Simon task when examining overall RTs, but that maximum deviations from the direct path to the correct response (see Figure 2) revealed the opposite—larger effects for the Simon task than the flanker. The critical difference here leading to the opposite outcomes is the temporal profile of the interference effects in each of the tasks. In the Simon task, the irrelevant location information leads to a strong automatic response, but this response occurs very early in the time course of the overall decision. In contrast, the interference is smaller for the flanker task, but overlaps more in time with the response selection process. Thus, mouse-tracking reveals information about the decision process and interference effects that are not present in examination of overall RTs that only measure discrete decisions. The authors also examined SCEs and reported better (albeit weak) within-task reliability for maximum deviation parameters than overall RTs. Relatedly, Ruitenberg et al. (2019) showed that within-task reliability of the item-specific proportion congruency effect (Jacoby et al., 2003), a theoretically similar observation to the SCE, is much higher (Bayes Factor BF_10_ = 610.02) for mouse-tracking movement times than for typical button presses (Bayes Factor BF_10_ = 10.43), again highlighting the differences in sensitivity between mouse-tracking and key presses. These findings collectively demonstrate the importance of examining continuous metrics of performance in addition to overall RT when assessing reliability of tasks.

To date, no studies, that the author is aware of, have examined between-task reliabilities of SCEs using mouse-tracking metrics beyond overall RTs as potentially more sensitive measures than overall RT on the mouse-tracking task. The present study examined the relationship between SCEs for the Stroop and the Flanker tasks using a mouse-tracking paradigm to assess the reliability and domain-generality or specificity of the effects.

## Materials and Methods

### Participants

Seventy undergraduate participants from Iowa State University participated in exchange for course credit. These data were collected as a part of an ongoing individual differences study that tackles a separate research question. Previous work examining the SCE with mousetracking on a Simon task revealed the effect size to be Cohen’s *d* = 0.71 for RT and *d* = 0.81 for area under the curve (Scherbaum and Kieslich, 2018). From this, the present study used the PANGEA online power analysis tool (Westfall, 2015; calculator available at https://jakewestfall.shinyapps.io/pangea/) to estimate the number of participants required to achieve at least 80% power with an alpha level set to 0.05 and the effect size to the more conservative *d* = 0.71 for the interaction term between current trial and previous congruency in the ANOVA in the present study. This analysis revealed that 28 participants would be required to detect the SCE with 80% power. Although one cannot completely equate the effect sizes for the Simon task with the Stroop and Flanker task used in the present study, it should be noted that the present study has 2.5 times the number of required participants suggested by the power analysis.

### Mouse-Tracking Task

The freely available software Mousetracker (Freeman and Ambady, 2010) was used to design the experiment and a Dell MS116 Wired Mouse was used to collect the data. Figure 2 depicts an example trial during the experiment. Trials began with two response boxes in the top left and right hand corners of the screen and a start button at the bottom center of the screen. Participants clicked the start button to begin the trial, after which a stimulus appeared in the center of the screen and participants were instructed to move their mouse as quickly and accurately as possible to the correct response box and click within the box. The response boxes remained on the screen for the duration of the trial. For the Flanker task, the stimuli were congruent or incongruent arrows (e.g., <<<<<OR<<><<, respectively) and participants were instructed to respond to the center arrow while ignoring the flanking arrows. For the Stroop task, the stimuli were congruent or incongruent colored words (e.g., the word BLUE printed in blue ink OR the word BLUE printed in red ink, respectively) and participants were instructed to respond to the ink color while ignoring what the word actually said. The stimulus remained on the screen until response selection. If participants took longer than 2,500 ms to response to the stimulus after appearance, a message upon response selection would appear, encouraging them to respond faster on the next trial. If an incorrect response was made, a red X would appear in place of the stimulus for 1,000 ms. Finally, the inter-stimulus interval was set to 1,000 ms. All participants completed 96 trials of the Stroop task and 96 trials of the Flanker task (order counterbalanced across participants).

The Mousetracking paradigm allows for the examination of several dependent variables (see right panel of Figure 2). Overall *Reaction Time* is the total time from click of the start button to click of the response box. *Initiation Time* is the time from click of the start button to initiation of mouse movement. *Movement Time* is the time after Initiation to completion (click) of response. Note that overall reaction time is simply the addition of movement time and initiation time. *Maximum Deviation* is the distance of maximum deviation from a straight line connecting the start point to the correct response box. Large maximum deviations are observed more often on incongruent trials than congruent trials because participants often start moving toward the incorrect response and overcorrect. *Time to Maximum Deviation* is the time from the beginning of the trial that it takes to reach the maximum deviation point. *Area Under the Curve* (AUC) is the area under the curve of the mouse movement trajectory relative to a straight line connecting the start point to the correct response. AUC always produces a positive value regardless of which side of the straight line the deviation occurs.

## Results

For transparency and reproducibility, all raw data, means, pivot tables, and files for the Mousetracker tasks used in the present manuscript are available for download via Figshare at the following links: Data, ^1^ Files for Mousetracker.^2^ All analyses were performed within the freely available Jamovi GUI (The Jamovi Project, 2021).

All analyses were performed on correct trials. Trials that followed errors were also removed. This is a standard procedure that removes the possibility that post-error adjustments are influencing the sequential adjustments of interest. There were very few errors overall, with only 1.2% of all trials for the flanker task and 2.6% of all trials for the Stroop task. The small number of errors prevented the examination of SCEs on error trials and post-error RTs that were initially of interest.

### Presence of Sequential Congruency Effect

**FIGURE 3:**
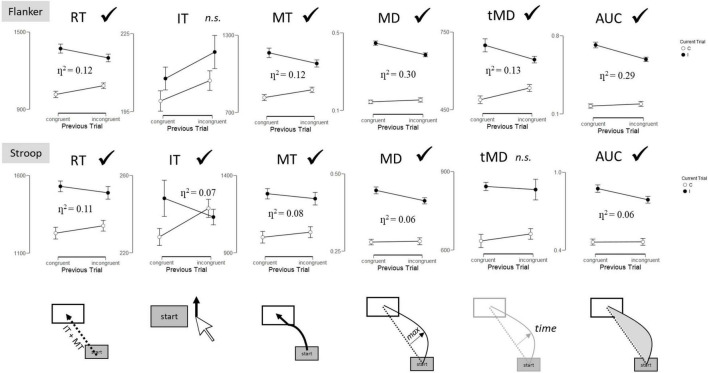
Sequential congruency effects (interaction between Current Trial and Previous Trial) by task and dependent variable. RT, Reaction Time; IT, Initiation Time; MT, Movement Time; MD, Maximum Deviation; tMD, Time to Maximum Deviation; AUC, Area Under the Curve. Checkmarks indicate significant effects (*p* < 0.05). n.s., non-significant.

Figure 3 illustrates the presence or absence of a significant SCE (interaction between current trial and previous trial) as a function of dependent variable for each task.

#### Flanker

##### Reaction Time

The 2 (Previous Trial: congruent, incongruent) × 2 (Current Trial: congruent, incongruent) ANOVA for overall Reaction Time (RT) revealed a significant effect of Current Trial, *F*(1, 69) = 60.06, *p* < 0.001, η^2^ = 0.47, with participants responding 286 ms faster to congruent than incongruent trials. A significant interaction between Current Trial and Previous Trial was also observed, *F*(1, 69) = 9.27, *p* = 0.003, η^2^ = 0.12. The interaction reveals the presence of the SCE, with smaller congruency effects following incongruent trials (216 ms) than congruent trials (355 ms).

##### Initiation Time

Initiation Times (IT) revealed a significant effect of Current Trial, *F*(1, 69) = 5.61, *p* = 0.02, η^2^ = 0.075, with participants responding 10 ms faster to congruent than incongruent trials. No other effects reached significance (*p* > 0.1).

##### Movement Time

Movement Times (MT) revealed a significant effect of Current Trial, *F*(1, 69) = 62.65, *p* < 0.001, η^2^ = 0.48, with participants responding 276 ms faster to congruent than incongruent trials. A significant interaction between Current Trial and Previous Trial was also observed, *F*(1, 69) = 9.35, *p* = 0.003, η^2^ = 0.12. The interaction reveals the presence of the SCE, with smaller congruency effects following incongruent trials (205 ms) than congruent trials (347 ms).

##### Maximum Deviation

Maximum Deviation (MD) revealed a significant effect of Current Trial, *F*(1, 69) = 289.7, *p* < 0.001, η^2^ = 0.81, with greater maximum deviation for incongruent than congruent trials. A significant interaction between Current Trial and Previous Trial was also observed, *F*(1, 69) = 29.2, *p* < 0.001, η^2^ = 0.30. The interaction reveals the presence of the SCE, with smaller congruency effects following incongruent than congruent trials.

##### Time to Maximum Deviation

Time to Maximum Deviation (tMD) revealed a significant effect of Current Trial, *F*(1, 69) = 52.89, *p* < 0.001, η^2^ = 0.43, with participants reaching maximum deviation 160 ms faster to congruent than incongruent trials. A significant interaction between Current Trial and Previous Trial was also observed, *F*(1, 69) = 10.13, *p* = 0.002, η^2^ = 0.13. The interaction reveals the presence of the SCE, with smaller congruency effects following incongruent trials (211 ms) than congruent trials (110 ms).

##### Area Under the Curve

Area Under the Curve (AUC) revealed a significant effect of Current Trial, *F*(1, 69) = 212.90, *p* < 0.001, η^2^ = 0.76, with greater area under the curve for incongruent than congruent trials. A significant interaction between Current Trial and Previous Trial was also observed, *F*(1, 69) = 28.76, *p* < 0.001, η^2^ = 0.29. The interaction reveals the presence of the SCE, with smaller congruency effects following incongruent than congruent trials.

#### Stroop

##### Reaction Time

The 2 (Previous Trial: congruent, incongruent) × 2 (Current Trial: congruent, incongruent) ANOVA for overall RT revealed a significant effect of Current Trial, *F*(1, 69) = 17.93, *p* < 0.001, η^2^ = 0.21, with participants responding 257 ms faster to congruent than incongruent trials. A significant interaction between Current Trial and Previous Trial was also observed, *F*(1, 69) = 8.32, *p* = 0.005, η^2^ = 0.11. The interaction reveals the presence of the SCE, with smaller congruency effects following incongruent (213 ms) than congruent trials (301 ms) (Figure 2).

##### Initiation Time

Initiation times revealed a significant interaction between Current Trial and Previous Trial, *F*(1, 69) = 5.13, *p* = 0.03, η^2^ = 0.07. The interaction reveals the presence of the SCE, with smaller congruency effects following incongruent (–4.5 ms) than congruent trials (20 ms).

##### Movement Time

Movement Times revealed a significant effect of Current Trial, *F*(1, 69) = 16.74, *p* < 0.001, η^2^ = 0.20, with participants responding 249 ms faster to congruent than incongruent trials. A significant interaction between Current Trial and Previous Trial was also observed, *F*(1, 69) = 5.97, *p* = 0.02, η^2^ = 0.08. The interaction reveals the presence of the SCE, with smaller congruency effects following incongruent trials (217 ms) than congruent trials (282 ms).

##### Maximum Deviation

Maximum Deviation revealed a significant effect of Current Trial, *F*(1, 69) = 116.26, *p* < 0.001, η^2^ = 0.63, with greater maximum deviation for incongruent than congruent trials. A significant interaction between Current Trial and Previous Trial was also observed, *F*(1, 69) = 4.65, *p* = 0.03, η^2^ = 0.06. The interaction reveals the presence of the SCE, with smaller congruency effects following incongruent than congruent trials.

##### Time to Maximum Deviation

Time to Maximum Deviation revealed a significant effect of Current Trial, *F*(1, 69) = 23.99, *p* < 0.001, η^2^ = 0.26, with participants reaching maximum deviation 189 ms faster to congruent than incongruent trials. No other effects reached significance (*p* > 0.26).

##### Area Under the Curve

Area Under the Curve revealed a significant effect of Current Trial, *F*(1, 69) = 109.68, *p* < 0.001, η^2^ = 0.61, with greater area under the curve for incongruent than congruent trials. A significant interaction between Current Trial and Previous Trial was also observed, *F*(1, 69) = 4.12, *p* = 0.05, η^2^ = 0.06. The interaction reveals the presence of the SCE, with smaller congruency effects following incongruent than congruent trials.

### Within-Task Split-Half Reliability of Sequential Congruency Effects

**FIGURE 4:**
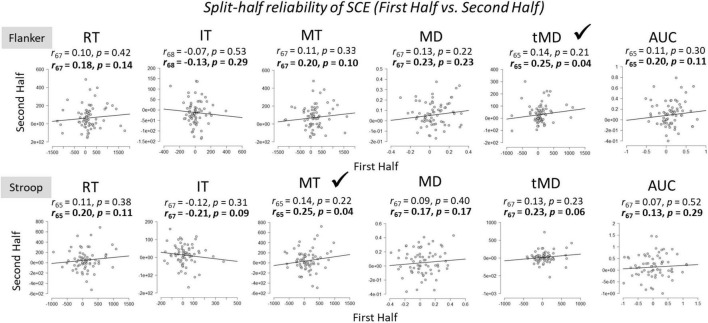
Within-task split-half (first half vs. second half of trials) reliabilities for Flanker and Stroop tasks. RT, Reaction Time; IT, Initiation Time; MT, Movement Time; MD, Maximum Deviation; tMD, Time to Maximum Deviation; AUC, Area Under the Curve. Significant reliability (i.e., positive Pearson *r* correlation coefficients) after Spearman-Brown prophecy correction are marked with a check mark. Un-bolded values above the graphs represent Pearson *r* correlation coefficients without correction, and bolded values represent Pearson *r* correlation coefficients after Spearman-Brown correction.

To assess the within-task reliability of SCEs, we correlated SCEs [(cI – cC) – (iI – iC)] in the first half of trials to SCEs in the second half of trials (Figure 4). Participants whose responses were more than 2 standard deviations above or below the group mean for each of the tasks were considered outliers and removed from the analyses. Initial results revealed that there was no relationship between the size of the SCE from the first half to the second half of the experiment for any of the dependent variables across both Flanker and Stroop tasks.

There are a few potential reasons for the low split-half within-task reliability estimates. First, only half of the trials are used in the reliability estimates using the split-half method. To correct for this, the present author used the Spearman-Brown’s prophecy formula (Brown, 1910; Spearman, 1910; see also de Vet et al., 2017 for a discussion of recent applications), outlined below:


$$2^{*}\,r/\left(1+r\right).$$

As can be seen from Figure 4, this correction did improve within-task reliability estimates, but reliability was still quite small and not statistically significant in several of the metrics.

**FIGURE 5:**
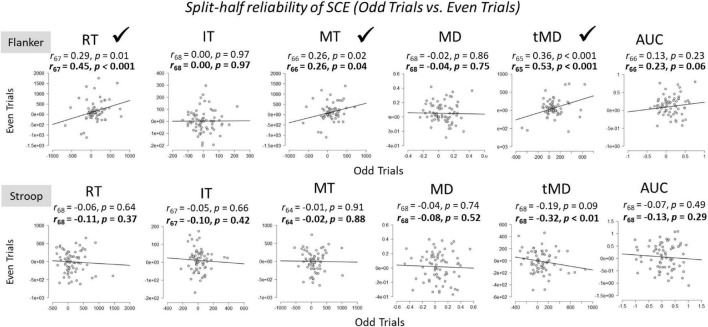
Within-task split-half (odd vs. even trials) reliabilities of the SCE for Flanker and Stroop tasks. RT, Reaction Time; IT, Initiation Time; MT, Movement Time; MD, Maximum Deviation; tMD, Time to Maximum Deviation; AUC, Area Under the Curve. Checkmarks indicate significant correlations (*p* < 0.05). Significant reliability (i.e., positive Pearson *r* correlation coefficients) after Spearman-Brown prophecy correction are marked with a check mark. Un-bolded values above the graphs represent Pearson *r* correlation coefficients without correction, and bolded values represent Pearson *r* correlation coefficients after Spearman-Brown correction.

A second potential reason for the low within-task reliability is practice from the first half of trials to the second half of trials. Following Whitehead et al. (2019), who argued that practice effects make the aforementioned split-half reliability difficult to interpret, we also examined split-half reliabilities across dependent variables by examining SCEs for odd trials compared to even trials (Figure 5). For the Stroop task, split-half reliabilities across all dependent variables were not significant. For the Flanker task, split-half reliabilities were weak, but significant, for overall Reaction Times, Movement Times, and Time to Maximum Deviation. These effects were again larger after Spearman-Brown correction. The within-task reliability for Area Under the Curve also approached (*p* = 0.06), but did not reach statistical significance, after Spearman-Brown correction. However, one could argue that these reliability estimates should be one-sided given that we expected only positive correlations, in which case, Area Under the Curve would be statistically reliable (*p* = 0.03).

### Between-Task Reliability of Sequential Congruency Effects

**FIGURE 6:**
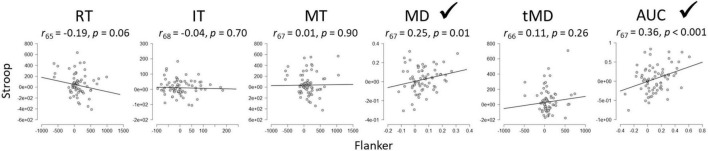
Between-task reliabilities of the SCE for Flanker and Stroop tasks. RT, Reaction Time; IT, Initiation Time; MT, Movement Time; MD, Maximum Deviation; tMD, Time to Maximum Deviation; AUC, Area Under the Curve. Checkmarks indicate significant correlations (*p* < 0.05).

Figure 6 illustrates the between-task reliability of SCEs for the Stroop and Flanker tasks Results revealed a significant correlation between the tasks for Maximum Deviation and Area Under the Curve, but *not* for the other dependent variables, including importantly, overall Reaction Times.

## Discussion

The present study examined the specificity and reliability of conflict adaptation effects using a mouse-tracking paradigm for the Stroop and Flanker tasks. Several important findings emerged. First, a significant sequential congruency effect (SCE) was observed on both Flanker and Stroop tasks across several mouse-tracking metrics. Second, SCEs showed weak or absent within-task reliability. Third, SCEs were correlated between tasks for mouse-tracking metrics that capture deviation from the optimal response path, but not for overall response times. These findings have implications for theories wishing to tease apart the specificity and generality of sequential congruency effects and demonstrate the power of examining dynamic responses in addition to discreet outcomes. Each finding is discussed in turn.

The finding that significant SCEs were observed for the both the Stroop and Flanker tasks is important given that it is the first report, that the author is aware of, to demonstrate SCEs with mouse-tracking on these tasks. Previous work has demonstrated robust SCEs on dynamic mouse-tracking metrics on the Simon task (Scherbaum et al., 2010, 2018), and the present findings contribute to this effort by examining dynamic processes of SCEs on two of the most commonly used paradigms in the literature: Stroop and Flanker. SCE effect sizes are often quite small (Braem et al., 2019), a finding replicated here with overall reaction times (η^2^ = 0.12). However, the effect sizes were substantially larger for measures of maximum deviation (η^2^ = 0.30) and area under the curve (η^2^ = 0.29) for the Flanker task. Thus, mouse-tracking paradigms might provide more sensitive measures of SCEs and allow more detailed investigations of conflict adaptation. On the other hand, the Stroop task did not replicate this pattern and the effect sizes were generally smaller than those observed on the Flanker task. This was not surprising given that van Steenbergen et al. (2015) demonstrated across four experiments that the Stroop task does not reliably demonstrate the SCE. They showed that this is due to the subjective experience of difficulty on the Stroop task relative to the flanker task. In the present study, the Stroop task was objectively more difficult, as evidenced by slower and less accurate responses than the flanker task. This may also help to explain the small within-task SCE reliability for the Flanker task but almost complete absence of reliability for the Stroop task (Figures 4, 5).

Using the most common method to estimate split-half reliability, we compared the first half of trials to the second half of trials within each task. Initial correlations revealed no significant split-half reliabilities on either task across all dependent variables. This replicates and extends recent findings by Whitehead et al. (2019) who also showed that Flanker and Stroop tasks showed no within-task reliability using this method. After Spearman-Brown correction to account for the fact that only half of the trials were being used for these estimates, reliability estimates were significant for Time to Maximum Deviation for the flanker task and Movement Times for the Stroop task, but *not* for overall Reaction Times. Accounting for practice effects by using odd trial compared to even trials produced significant split-half within-task reliability for four out of the six metrics on the Flanker task, which is the task that generally showed stronger SCEs. Furthermore, the strongest within-task reliability estimate was observed on the Time to Maximum Deviation metric. The Stroop task did not produce any significant within-task correlations among the dependent variables. Overall, despite showing large SCEs for both the Flanker and the Stroop task at the group level across dependent variables, within-task reliabilities were weak at best, and absent in many correlations. These findings are problematic for researchers wishing to use an individual differences approach on conflict adaptation effects, and similar issue have been raised for overall congruency effects (Rouder and Haaf, 2019).

The central issue of the present paper however was to assess the generalizability of conflict adaptation effects across rather than within tasks. In other words, to the extent that an individual shows a large SCE on one task, will he/she/they show a large effect on the other? The answer to this question has implications for theories like the conflict monitoring theory (Botvinick et al., 2001), one of the most influential theories of cognitive control to date (Schmidt, 2019). The theory stipulates that a general control mechanism is responsible for conflict adaptation, and thus, SCEs should correlate across tasks (Whitehead et al., 2019). Using overall reaction times in the present experiment, as has been done previously (reviews in Egner, 2014; Schmidt, 2019), the observed outcome is that SCEs do not correlate across tasks, which is problematic for the conflict monitoring theory. However, maximum deviation and area under the curve of the mouse trajectory, reflecting dynamic decision processes before response execution, revealed significant (albeit weak) between-task reliability.

It is important to note that the present findings are not meant to provide definitive evidence for or against the conflict monitoring theory, nor can they speak to the longstanding debate between top-down (Botvinick et al., 2001; Clayson and Larson, 2011; Weissman et al., 2014; Feldman and Freitas, 2016) and bottom-up (Hommel et al., 2004; Schmidt and De Houwer, 2011; Schmidt, 2019) accounts of the SCE. This is especially true considering that the present design contains feature/response repetitions and contingency learning confounds. There were unfortunately not enough trials in the present study to examine response repetitions needed to tease apart these theories, but future studies are encouraged to do so. More trials would also allow for researchers to examine better estimates for both within- and between-task reliability estimates. It is important to note that within- and between-task reliability estimates should not be treated as completely separate phenomena given that one should not expect between-task reliability if within-task reliability estimates are not significant. A reason for any observation in which within-task reliability is weak or absent and between-task reliability is significant can be explained by the number of trials within each of the calculations. The within-task reliability is calculated by correlating either odd trials to even trials or the first half of trials to second half of trials. The between task reliability is calculated by correlating the overall SCE across all trials. This means that the within-task reliability estimates use only half the number of trials that the between task reliability uses, which lowers the reliability estimate by design. Another issue is that trial numbers in the present study are already lower than the number of trials for reliability estimations on typical Stroop and Flanker tasks, given that the task was initially designed for a separate purpose (as stated on page 6 under the Participants subheading). The SCE paradigm in the present paper deviates from the usual SCE paradigm for button presses in that there is a start button at the beginning of every trial. This could contribute to the observed reliability effects. A final consideration is that some have argued that experimental tasks are not designed to be used as individual differences measures, so it may be the case that a quest for more sensitive measures of SCEs is not as meaningful as one might hope (Hedge et al., 2018; Luck, 2019).

Despite the aforementioned limitations, Friedman and Miyake (2017) have made the argument that the latent factor behind the tasks measuring inhibition is quite reliable despite separate-task indices not exhibiting sufficient internal reliability. Nonetheless, given the above limitations, the present data and findings should be treated as preliminary. Future research should add more trials and replicate the initial findings to gain more confidence. Future studies might also consider combining the Stroop and Flanker tasks within the same block to investigate the stability of SCEs between tasks directly.

In sum, the main contribution of the present study was mainly methodological, and illustrates that simple button presses might not be sensitive enough on their own to determine the specificity and/or generalizability of sequential congruency effects. Three main findings emerged: (1) Robust SCEs were observed for both the Stroop and Flanker tasks at the group level, (2) Within-task split-half reliabilities for the SCE across dependent variables were weak at best and non-existent in many cases, and (3) SCEs for the Flanker and Stroop tasks did not correlate with each other for overall reaction times, but did show significant correlations between tasks on more dynamic measures that captured processes before response execution. The findings suggest that future studies examining these questions should employ mouse-tracking paradigms with carefully controlled conditions to tease apart the theories.

## Data Availability Statement

The datasets presented in this study can be found in online repositories. The names of the repository/repositories and accession number(s) can be found below: Data, https://figshare.com/s/6328976673f692673031; Files for Mousetracker, https://figshare.com/s/c400f6f726d7e85ff989.

## Ethics Statement

The studies involving human participants were reviewed and approved by Institutional Review Board (IRB), Iowa State University. The patients/participants provided their written informed consent to participate in this study.

## Author Contributions

JG conceptualized, designed, performed statistical analyses, and wrote the manuscript.

## Conflict of Interest

The author declares that the research was conducted in the absence of any commercial or financial relationships that could be construed as a potential conflict of interest.

## Publisher’s Note

All claims expressed in this article are solely those of the authors and do not necessarily represent those of their affiliated organizations, or those of the publisher, the editors and the reviewers. Any product that may be evaluated in this article, or claim that may be made by its manufacturer, is not guaranteed or endorsed by the publisher.

## Footnotes

1
https://figshare.com/s/6328976673f692673031

2
https://figshare.com/s/c400f6f726d7e85ff989
